# Effect of Light Irradiance and Curing Duration on Degree of Conversion of Dual-Cure Resin Core in Various Cavities with Different Depths and Diameters

**DOI:** 10.3390/ma17174342

**Published:** 2024-09-02

**Authors:** Ho-Kyung Lim, Subramanian Keerthana, So-Yeon Song, Chongyang Li, Ji Suk Shim, Jae Jun Ryu

**Affiliations:** 1 Department of Oral and Maxillofacial Surgery, Korea University Guro Hospital, 148, Gurodong-ro, Guro-gu, Seoul 08308, Republic of Korea; ungassi@naver.com; 2 Graduate School of Clinical Dentistry, Korea University, 46, Gaeunsa 2-gil, Seongbuk-gu, Seoul 02842, Republic of Korea; drkeerthanasubramani@gmail.com (S.K.); 18841134423@163.com (C.L.); 3 Department of Prosthodontics, Korea University Guro Hospital, 148, Gurodong-ro, Guro-gu, Seoul 08308, Republic of Korea; ssongchu2@gmail.com; 4 Department of Prosthodontics, Korea University Anam Hospital, 73, Goryeodae-ro, Seongbuk-gu, Seoul 02841, Republic of Korea

**Keywords:** dual-core resin, degree of conversion, light irradiance, curing duration, cavity size

## Abstract

(1) Background: To compare the degree of conversion of resin cores in various types of cavities and determine an effective irradiation method for achieving a higher degree of conversion. (2) Methods: Four different-sized cavities (narrow–shallow, narrow–deep, wide–shallow, and wide–deep) were simulated using a Teflon mold. The light irradiance reaching the bottom of each mold was measured by positioning a radiometer. The degree of conversion of the dual-cure resin core after irradiation (400 mW/cm^2^ for 20 s, 400 mW/cm^2^ for 40 s, and 800 mW/cm^2^ for 20 s) was measured using Fourier-transform near-infrared spectroscopy. (3) Results: The highest light irradiance was found at the bottom of wide–shallow cavities, followed by wide–deep, narrow–shallow, and narrow–deep ones (*p* < 0.001). In narrow cavities, irradiation at 800 mW/cm^2^ for 20 s led to a significantly higher degree of conversion (*p* < 0.001). In wide cavities, irradiation at 400 mW/cm^2^ for 40 s and 800 mW/cm^2^ for 20 s both led to a significantly higher degree of conversion (*p* < 0.001). (4) Conclusions: Less curing light reaches the bottom of cavities with a smaller diameter and greater depth. Providing a higher irradiance of light can induce a higher degree of conversion of resin composites in narrower cavities.

## 1. Introduction

The core is defined as the foundation restoration which restores a sufficient amount of the coronal anatomy of a vital or endodontically treated tooth [1]. The required characteristics of a desirable core are adequate compressive strength to resist forces, high flexural strength, the ability to bond to the remaining tooth structure, biocompatibility, and ease of handling [2]. Resin composite is one of more popular materials used for core build-up, because it has good mechanical characteristics and low solubility, is able to bond to dentin, and is easy to manipulate. In addition, resin composite can provide an esthetic base under all ceramic restorations [3].

A high degree of conversion (DC) of composite polymerization is a prerequisite for obtaining the optimal physical and mechanical properties, biocompatibility, and improved success of composite restoration [4,5]. To ensure sufficient DC, it is beneficial to use a dual-cure resin composite as the core material [6]. Less curing light reaches the bottom of the core material in a cavity because the light curing is applied from the top of the core material to the bottom, and the light irradiance of a light-curing unit decreases depending on the distance from the light-curing tip [7]. The dual-cure resin composite is a resin that uses both photo-polymerization and auto-polymerization mechanisms to be cured [8]; areas that undergo less light curing are mainly polymerized through auto-polymerization.

However, less polymerization may occur at the bottom of the dual-cure resin core material than at the top. Although auto-polymerization is guaranteed, adequate light curing is essential in order to achieve the highest DC of dual-cure resins [9]. When light is irradiated into the composite restoration, the light irradiance is decreased because of light scattering, absorption, and reflectance, which are major factors associated with light attenuation [10,11]. As the thickness of the material increases, light penetration through the resin restorative material also decreases, resulting in a lower DC [12]. Therefore, the shape of the cavity filled by the core material affects the DC of the dual-cure resin core.

To compensate for the decrease in light irradiance at the bottom of the core, modifying various light-curing-related factors have been considered, including the light source, light intensity, and curing duration. The depth of the restoration reached by enough irradiance is known as the depth of cure. Previous studies have compared the depth of cure between single light-emitting diode (LED) light-curing units (LCUs), and halogen LCUs showed that LED units demonstrated a greater depth of cure than that of halogen units [13,14]. A higher light irradiance is also related to a greater depth of cure. Moreover, a higher irradiance is applicable because LED units recently released in the market deliver much higher light irradiances, greater than 2000 mW/cm^2^. For providing more exposure time from curing light, increasing the curing duration can also be an additional strategic approach [15].

The purpose of this study was to evaluate the effects of light intensity and curing duration on the DCs of dual-cure resin cores in various cavities with different diameters and depths. The researcher’s hypothesis is that there is a difference in DC according to the light intensity and duration, and various cavities with different diameters and depths, and the null hypothesis is the opposite, that there is no difference in DC according to light intensity and duration, and various cavities with different diameters and depths. Dual-cure resin cores were placed in four differently simulated cavities (diameter × depth: 3 mm × 5 mm, 3 mm × 10 mm, 5 mm × 5 mm, and 5 mm × 10 mm). Various light intensities and curing durations were applied to the resin cores, and the DC was evaluated.

## 2. Materials and Methods

### 2.1. Teflon Mold Fabrication

**Figure 1:**
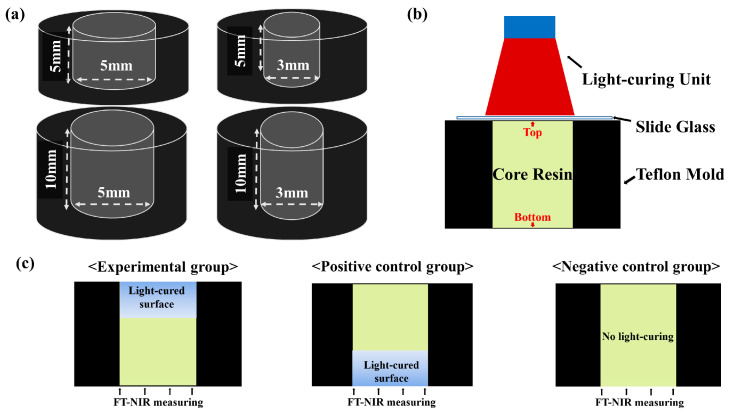
The schematic diagram of the study design. (**a**) Teflon molds were developed to simulate narrow and shallow cavity (NS, 3.0 mm in diameter and 5.0 mm in depth), narrow and deep cavity (ND, 3.0 mm in diameter and 10.0 mm in depth), wide and shallow cavity (WS, 5.0 mm in diameter and 5.0 mm in depth), and wide and deep cavity (WD, 5.0 mm in diameter and 10.0 mm in depth). (**b**) After injecting dual-cure resin into the prepared Teflon mold, light curing was performed. (**c**) Unlike the experimental group, positive control was defined as specimens that were light-cured from the bottom, not the top, and that the same surface was exposed to curing light and measured for DC. Moreover, negative control was defined as specimens that were not exposed to any light.

Figure 1 shows the shape of the Teflon mold and the methods used to prepare the specimens. The cross-sectional shape of the core was circular, and the overall shape was formed to have a cylindrical shape. The size of the hole in the resin core was determined considering the anatomic features of the premolars and molars. Although there was no reference for the average size of the cavity for the core, the diameter of the core should be smaller than the mesio-distal width of the tooth. Previous studies showed that the average mesio-distal width of a crown is approximately 10 mm for molars and 6.5 mm for premolars [16,17]. The depth of the cavity can be estimated by adding the length of remaining crown and root trunk and varies more than the diameter of the core because root trunks show a variety in length, ranging approximately 2–9 mm for molars and 4–13 mm for premolars [18]. Referring to the anatomic features of the tooth, four types of Teflon molds were developed to simulate a narrow and shallow cavity (NS, 3.0 mm in diameter and 5.0 mm in depth), narrow and deep cavity (ND, 3.0 mm in diameter and 10.0 mm in depth), wide and shallow cavity (WS, 5.0 mm in diameter and 5.0 mm in depth), and wide and deep cavity (WD, 5.0 mm in diameter and 10.0 mm in depth).

### 2.2. Light Curing of Various Teflon Molds and Measuring Light Irradiance at the Bottom of Teflon Mold

In this study, in order to exclude the effect of the various wavelengths of the LED LCU, a second-generation photo-polymerizer composed of a monowave single LED was used. The output of a single LED light LCU (Dr’s Light, Good Doctors Co., Incheon, Republic of Korea) was manually set up to emit two levels of light irradiance, 400 and 800 mW/cm^2^, which were the maximum and minimum intensity values provided by the curing unit manufacturer. The minimum light irradiance (400 mW/cm^2^) and curing duration (20 s) were obtained from the manufacturer’s instructions for the resin core. The light intensities reaching the bottom of the Teflon mold were measured without the resin core material. Light curing at an irradiance of 400 or 800 mW/cm^2^ for 20 s was applied from the top of the Teflon mold, and the irradiance was measured using a radiometer (LED radiometer; Good Doctors Co., Incheon, Republic of Korea) at the bottom of the Teflon mold. The LED LCU emitted blue light, and the wavelength range was 380–500 nm. The diameter of the LCU tip was 1 cm, wider than the diameter of the mold, so that the light source could cover the entire resin in the Teflon mold. The sensor size of the LED radiometer was the same as that of the Teflon mold.

### 2.3. Preparation of Specimen for Measuring DC

Table 1 shows the details of the specimens prepared for DC measurement. Each specimen was filled with Luxacore DC (DMG, Hamburg, Germany; A2, Lot 590488), a light-curing resin core widely used in clinical practice, containing camphorquinone as a photo-initiator. The core resins were mixed with auto-mixing tips inserted into a Teflon mold. Excess cement was removed by placing transparent microscopic cover slips. A single LED light-curing unit was manually calibrated, varying the pulse width of LED using a microcontroller controlling the duty cycle of LED. The calibrated irradiances were confirmed with a radiometer. The LCU was set up to emit two different light irradiances and the irradiances were verified 10 times as follows: 397.3 ± 3.5 mW/cm^2^ (≒400 mW/cm^2^) and 803.4 ± 5.2 mW/cm^2^ (≒800 mW/cm^2^). Referred from the recommendation of manufacturer of Luxacore DC, the minimal irradiation and curing duration was determined. With two irradiances and two curing durations, three different irradiation protocols were determined: minimal irradiation (400 mW/cm^2^, 20 s), irradiation with longer curing duration (400 mW/cm^2^, 40 s), and irradiation with higher light irradiance (800 mW/cm^2^, 20 s). Negative control was defined as specimens that were not exposed to any light. Moreover, positive control was defined as specimens that were light-cured from the bottom, not the top, and that the same surface was exposed to curing light and measured for DC.

### 2.4. Measuring the DC

After waiting for a chemical curing time of 5 min, as suggested by the manufacturer, the DC of each resin core was evaluated using Fourier-transform near-infrared spectroscopy (FT-NIR; NIRSolutions^TM^, BUCHI, Flawil, Switzerland). Except for the positive control group, the DC was measured from the bottom surface of the specimens (i.e., the top surface was exposed to curing light; thus, it was assumed that complete polymerization was achieved on the top surface). Absorbance spectra were measured by scanning the specimens 10 times over a range of 4000–8000 cm^−1^, with a resolution of 4 cm^−1^. To calculate the DC, the area of the peak at 6165 cm^−1^, which corresponds to vinyl stretching, was used [19]. The DC was acquired by calculating the ratio of the peak area in the monomeric to polymeric states.

### 2.5. Statistical Analysis

The mean and standard deviation for light irradiance and DC were obtained for each group. Since the investigated data did not show normality in the normality test, a nonparametric test was used to compare groups. We used the Kruskal–Wallis test to evaluate the effect of cavity type on light irradiance, and the Mann–Whitney U test was used to evaluate the effect of the three different irradiation protocols on light irradiance. Statistical differences between groups were determined using the Dwass, Steel, and Critchlow–Fligner methods. The effects of irradiation protocols and cavity types on DC were evaluated using one-way ANOVA and Tukey’s multiple comparison tests. Statistical significance was set at *p* < 0.05. All statistical analyses were performed using SAS software (version 9.4; SAS Institute Inc., Cary, NC, USA).

## 3. Results

Table 2 shows the light intensities reaching the bottom of each Teflon mold when light-cured from the top of Teflon molds using 400 or 800 mW/cm^2^ irradiation. In the same Teflon mold type, light exposure at 800 mW/cm^2^ showed a higher light irradiance than 400 mW/cm^2^, with statistical significance (*p* < 0.001). At the same irradiance, a higher light irradiance was shown in the order of WS, WD, NS, and ND, with statistical significance (*p* < 0.001).

Table 3 shows the effect of the irradiance protocols and Teflon mold sizes on the degree of conversion. Irradiating with 400 mW/cm^2^ for 20 s led to a significantly lower DC than that with 400 mW/cm^2^ for 40 s and 800 mW/cm^2^ for 20 s (*p* < 0.001). There was no statistical difference between the DC caused by irradiation at 800 mW/cm^2^ for 20 s and 400 mW/cm^2^ for 40 s (*p* > 0.05). Wide molds (WS and WD) showed a higher DC than shallow molds (NS and ND) (*p* < 0.001). Shallow molds with a wide diameter (WS) showed a higher DC than deep molds with a wide diameter (WD) (*p* < 0.001), but there was no statistical difference between shallow molds with a narrow diameter (NS) and deep molds with a narrow diameter (ND) (*p* > 0.05).

Table 4 shows the DC of each group, and a statistical comparison between the DCs caused by various irradiation protocols. For NS and ND cavities, irradiation with negative control with 400 mW/cm^2^ for 20 s and with 400 mW/cm^2^ for 40 s were not significantly different (*p* > 0.05). The positive control showed the highest DC, and irradiation at 800 mW/cm^2^ for 20 s led to a significantly higher DC than that achieved using the other irradiation protocols in NS and ND cavities (*p* < 0.001). For the WS and WD cavities, the positive control achieved the highest DC, followed by irradiation at 800 mW/cm^2^ for 20 s, 400 mW/cm^2^ for 40 s, 400 mW/cm^2^ for 20 s, and the negative control. There was no significant difference in DC between that achieved with irradiation at 800 mW/cm^2^ for 20 s and 400 mW/cm^2^ for 40 s (*p* > 0.05); however, the other groups were significantly different (*p* < 0.001).

## 4. Discussion

Inadequate polymerization is associated with the unfavorable results of resin composites, including a higher solubility, inferior physical properties, and unfavorable pulpal effects [20,21,22]. As less curing light approaches the floor of the cavity, a high DC is hard to achieve at the bottom of the resin core. The researcher’s hypothesis is that there is a difference in DC according to the light intensity and duration, and various cavities with different diameters and depths, and the null hypothesis is the opposite, that there is no difference in DC according to the light intensity and duration, and various cavities with different diameters and depths. The present study aimed to compare the DCs of resin cores in various types of cavities and determine effective irradiation protocols for achieving a higher DC of the resin core. To evaluate the effect of the diameter and depth of the cavity on the approached light irradiance and the DC of resin composites at the bottom of the cavity, four cavity types were used, in which two diameters and two depths were combined. To evaluate the effect of light irradiance and exposure duration, three irradiation protocols were assigned to the resin core, including irradiation following the manufacturer’s instructions, irradiation for longer exposure durations, and irradiation with a higher intensity. The results show that less light approached the bottom of the cavity with a shorter diameter and deeper depth. To achieve a higher DC, a higher light intensity and longer exposure duration were valid for the molar groups, but a longer exposure duration does not seem to be effective in the narrow cavities.

The present study shows that light irradiance at the bottom of the cavity is affected by the shape of the cavity. The results demonstrate that a deeper and narrower cavity is associated with a lower light irradiance at the bottom, and that special light-curing strategies are necessary to achieve a high DC. The result that a deeper cavity is related to lower light irradiance is a predictable outcome that has been proven in multiple previous studies, because the distance from the tip of the LCU is inversely proportional to light irradiance [23,24,25]. However, the present study showed lower light irradiance at the bottom of the cavity than that reported in previous studies. A previous study showed that the light irradiance fell to 50% at 6 mm [25], that approximately 20% of the total light irradiance was lost for every 1 mm of air space [23]. In this study, there was a decrease in light irradiance of more than 85% at the bottom of the cavity with a depth of 5.0 mm. Regarding the result that lower light irradiance reached the bottom of the narrower cavity, previous studies showed different results depending on the backing reflectance of the mold [26]. Similar results to those of this study were obtained in studies that used dark-colored polymers as the mold [27]; however, opposite results were obtained in studies using brightly colored stainless steel [28].

In this study, positive control refers to the group with the highest DC that underwent the appropriate light curing following the manufacturer’s instructions. The result where all groups had a lower DC than that of the positive control group shows that none of the groups achieved the DC recommended by the manufacturer. This means that a higher light irradiance or longer exposure duration should be applied to the resin core than that used in this study for all types of cavities. For adequate polymerization, three factors of light curing should be appropriately modified, including the wavelength of the light source, irradiance, and curing duration [29,30]. Under the premise that an effective wavelength for the photo-initiator of resin composite is used, previous studies have suggested guidelines for adequate light irradiance and curing duration. Rueggeberg et al. showed that an irradiance of 400 mW/cm^2^ for 40 s is necessary for resin composites with a thickness of 2 mm [31]. Manga et al. suggested irradiation at 600 mW/cm^2^ for 40 s for resin composites of 2 mm thickness [32]. Leonard et al. recommended providing a minimum irradiance of 300 mW/cm^2^ for a micro-filled resin composite of 2 mm thickness [29]. As the thickness of the resin was more than 5 mm in this study, irradiating at 800 mW/cm^2^ for 40 s was not sufficient to cause maximal photo-polymerization.

The negative control group was dual-resins not treated by any curing light and had the lowest DC among groups. The application of 400 mW/cm^2^ in the NS and ND groups led to statistically similar results to those of the negative control group. In addition, increasing the curing time also did not affect the DC in the NS and ND groups at 400 mW/cm^2^ irradiance. This means that a light irradiance of 400 mW/cm^2^ did not result in any photo-polymerization at the bottom of NS and ND cavities. To compensate for irradiation at lower light intensities, light exposure for longer durations can be used [15,33,34], but it is an effective method under the condition that sufficient light irradiance is provided to activate the photo-initiator. In this study, a light intensity valid for photo-polymerization can be distinguished from the results whether the exposure duration affects the DC with a specific light intensity. The minimal irradiance affecting photo-polymerization was 64 mW/cm^2^ at the bottom of the mold. For the molar groups (WS and WD), the two irradiation protocols of irradiating at 400 mW/cm^2^ for 40 s and irradiating 800 mW/cm^2^ for 20 s led to statistically the same DC. However, for the premolar groups (NS and ND), irradiating at 800 mW/cm^2^ for 20 s led to a statistically higher DC than irradiation at 400 mW/cm^2^ for 40 s. These results suggest that providing a higher intensity of light is recommended to induce an appropriate DC of resin composites in narrow cavities.

Although this study confirmed that a high irradiance in a narrow and deep cavity is more effective in inducing a high DC than a long duration, in actual clinical practice, pulp vitality affected by the temperature change due to light curing cannot but be considered. It is known that an increase of 5.5 °C can cause 15% of pulp damage, and an increase of 11 °C can cause 60% of pulp damage [35]. In one study investigating the temperature change in the composite by controlling the time and duty ratio of the LCU, the higher the duty ratio is, the more the temperature increase was observed [36]. It was mentioned that, in order to prevent a sudden increase in temperature, polymerization should be carried out while gradually decreasing the intensity or by adjusting the angle of the polymerization reactor [36]. It is thought that further research is needed in the future on the content related to the increase in temperature.

In this study, a single LED LCU was used, and the intensity of the LCU was manually controlled using a radiometer. Measurement using a single light source is essential in order to draw concise conclusions for comparing the effects of light intensity, because polymerization kinetics can be affected by the type of LCU [37]. However, the differences between the enamel–dentin wall and white colored Teflon may lead to different results because the depth of cure is affected by the characteristics of the material that constitutes the mold [26]. In addition, further studies with various resin composites may be necessary because the type of resin and color of the material can affect the irradiance of light exposure [38].

In summary, As the light intensity and duration increased, the DC increased, and, as the cavity diameter narrowed and depth increased, the DC decreased. This suggests that, the narrower and deeper the cavity is, the more the polymerization energy or time must be increased to reach the same DC. Although the authors have obtained significant insights into the DC according to the cavity diameter and depth, further research is needed to clarify the in vivo environment and to investigate various light sources or materials.

## 5. Conclusions

Less curing light could reach the bottom of cavities with a smaller diameter and greater depth. To achieve a high DC of resin composites in a narrower and deep cavity, providing a higher irradiance of light might be appropriate. However, this is based entirely on current laboratory studies, and further studies are needed to support these conclusions.

## Figures and Tables

**Table 1:** Study design.

Cavity Type (Diameter × Length) /Irradiating Methods (Intensity, Duration)	Low Intensity/Short Duration (L20, 400 mW/cm^2^, 20 s)	Low Intensity/Long Duration (L40, 400 mW/cm^2^, 40 s)	High Intensity/Short Duration (H20, 800 mW/cm^2^, 20 s)
Narrow/Shallow (NS, Ø 3.0 × 5.0 mm)	NS-L20 (N = 12)	NS-L40 (N = 12)	NS-H20 (N = 12)
Narrow/Deep (ND, Ø 3.0 × 10.0 mm)	ND-L20 (N = 12)	ND-L40 (N = 12)	ND-H20 (N = 12)
Wide/Shallow (WS, Ø 5.0 × 5.0 mm)	WS-L20 (N = 12)	WS-L40 (N = 12)	WS-H20 (N = 12)
Wide/Deep (WD, Ø 5.0 × 10.0 mm)	WD-L20 (N = 12)	WD-L40 (N = 12)	WD-H20 (N = 12)

N = number of specimens.

**Table 2:** The light intensities at the bottom of each Teflon molds exposed to 400 or 800 mW/cm^2^.

	N	Mean (SD)	Median (IQR)	*p*-Value *	*p*-Value ^†^
400 mW/cm^2^				<0.001	<0.001
Narrow/Shallow	10	29.2 (3.1)	29.0 (27.0, 31.0)	b	a
Narrow/Deep	10	21.5 (3.0)	21.0 (19.0, 22.0)	a	a
Wide/Shallow	10	83.9 (6.5)	83.5 (78.0, 87.0)	d	a
Wide/Deep	10	64.8 (4.6)	64.5 (61.0, 69.0)	c	a
800 mW/cm^2^					
Narrow/Shallow	10	63.4 (5.9)	64.5 (62.0, 67.0)	b	b
Narrow/Deep	10	47.7 (2.8)	48.5 (45.0, 50.0)	a	b
Wide/Shallow	10	184.7 (5.1)	187.0 (183.0, 188.0)	d	b
Wide/Deep	10	141.7 (5.8)	143.0 (141.0, 146.0)	c	b

N: number, SD: standard deviation, IQR: interquartile range; *: statistical differences between resin cores in different cavity types, Kruskal–Wallis Test; ^†^: statistical differences between resin cores applied different light intensities, Mann–Whitney U Test; a, b, c, d: The different alphabets represent that the groups are significantly different from each other by the Dwass, Steel, and Critchlow–Fligner methods.

**Table 3:** The effect of irradiance methods and Teflon mold sizes on the degree of conversion.

	N	Mean (SD)	Median (IQR)	*p*-Value *
**Irradiance**				<0.001
400 mW/cm^2^, 20 s	48	43.8 (4.7)	44.4 (40.2, 47.3)	a
400 mW/cm^2^, 40 s	48	49.3 (10.0)	48.8 (41.7, 59.0)	b
800 mW/cm^2^, 20 s	48	52.7 (7.6)	51.7 (46.5, 59.5)	b
Teflon Mold				<0.001
Narrow/Shallow	36	42.6 (4.2)	42.9 (39.3, 45.5)	a
Narrow/Deep	36	41.9 (4.4)	41.7 (39.4, 44.8)	a
Wide/Shallow	36	57.1 (7.2)	59.3 (50.6, 62.9)	c
Wide/Deep	36	52.8 (5.5)	52.1 (48.0, 56.8)	b

N: number, SD: standard deviation, IQR: interquartile range; *: *p*-value by ANOVA Test; a, b, c: The different alphabets represent that the groups are significantly different from each other by Tukey’s test.

**Table 4:** The degree of conversion by different irradiating methods in various Teflon mold sizes.

	Mean (SD)	Median (IQR)	*p*-Value *
**Narrow/Shallow**			<0.001
Negative control	39.9 (2.5)	39.2 (38.0, 42.4)	a
400 mW/cm^2^, 20 s	39.9 (2.6)	40.2 (38.0, 40.5)	a
400 mW/cm^2^, 40 s	40.9 (3.5)	42.1 (37.0, 43.6)	a
800 mW/cm^2^, 20 s	46.9 (2.8)	47.0 (44.0, 50.1)	b
Positive control	72.3 (5.3)	73.8 (67.3, 76.7)	c
Narrow/Deep			<0.001
Negative control	39.9 (2.5)	39.2 (38.0, 42.4)	a
400 mW/cm^2^, 20 s	40.2 (3.8)	40.8 (37.3, 42.6)	a
400 mW/cm^2^, 40 s	39.7 (3.5)	39.8 (37.5, 41.7)	a
800 mW/cm^2^, 20 s	45.9 (3.0)	46.0 (43.4, 47.8)	b
Positive control	72.3 (5.3)	73.8 (67.3, 76.7)	c
Wide/Shallow			<0.001
Negative control	39.9 (2.5)	39.2 (38.0, 42.4)	a
400 mW/cm^2^, 20 s	48.7 (2.5)	49.0 (46.5, 50.6)	b
400 mW/cm^2^, 40 s	60.9 (4.4)	59.5 (58.4, 65.5)	c
800 mW/cm^2^, 20 s	61.8 (4.6)	61.6 (60.3, 64.6)	c
Positive control	72.3 (5.3)	73.8 (67.3, 76.7)	d
Wide/Deep			<0.001
Negative control	39.9 (2.5)	39.2 (38.0, 42.4)	a
400 mW/cm^2^, 20 s	46.5 (2.2)	46.9 (45.4, 48.0)	b
400 mW/cm^2^, 40 s	55.8 (3.4)	56.1 (52.1, 59.1)	c
800 mW/cm^2^, 20 s	56.3 (3.8)	56.0 (53.2, 58.6)	c
Positive control	72.3 (5.3)	73.8 (67.3, 76.7)	d

SD: standard deviation, IQR: interquartile range; *: *p*-value by ANOVA Test; a, b, c, d: The different alphabets represent that the groups are significantly different from each other by Tukey’s test.

## Data Availability

The datasets used during the current study are available from the corresponding author upon reasonable request.
